# Berberine Could Ameliorate Cardiac Dysfunction via Interfering Myocardial Lipidomic Profiles in the Rat Model of Diabetic Cardiomyopathy

**DOI:** 10.3389/fphys.2018.01042

**Published:** 2018-08-02

**Authors:** Shifen Dong, Shuofeng Zhang, Zhirong Chen, Rong Zhang, Linyue Tian, Long Cheng, Fei Shang, Jianning Sun

**Affiliations:** ^1^School of Chinese Materia Medica, Beijing University of Chinese Medicine, Beijing, China; ^2^Department of Pharmacology, Analysis and Testing Center, Beijing University of Chemical Technology, Beijing, China

**Keywords:** diabetic cardiomyopathy, myocardial tissue, lipidomic profiles, berberine, UPLC/Q-TOF/MS

## Abstract

**Background:** Diabetic cardiomyopathy (DCM) is considered to be a distinct clinical entity independent of concomitant macro- and microvascular disorders, which is initiated partly by disturbances in energy substrates. This study was to observe the dynamic modulations of berberine in DCM rats and explore the changes of lipidomic profiles of myocardial tissue.

**Methods:** Sprague-Dawley (SD) rats were fed high-sucrose and high-fat diet (HSHFD) for totally 22 weeks and intraperitoneally (i.p.) injected with 30 mg/kg of streptozotocin (STZ) at the fifth week to induce DCM. Seventy-two hours after STZ injection, the rats were orally given with berberine at 10, 30 mg/kg and metformin at 200 mg/kg, respectively. Dynamic changes of cardiac function, heart mass ratios and blood lipids were observed at f 4, 10, 16, and 22, respectively. Furthermore, lipid metabolites in myocardial tissue at week 16 were profiled by the ultra-high-performance liquid chromatography coupled to a quadruple time of flight mass spectrometer (UPLC/Q-TOF/MS) approach.

**Results:** Berberine could protect against cardiac diastolic and systolic dysfunctions, as well as cardiac hypertrophy, and the most effective duration is with 16-week of administration. Meanwhile, 17 potential biomarkers of phosphatidylcholines (PCs), phosphatidylethanolamines (PEs) and sphingolipids (SMs) of DCM induced by HSFD/STZ were identified. The perturbations of lipidomic profiles could be partly reversed with berberine intervention, i.e., PC (16:0/20:4), PC (18:2/0:0), PC (18:0/18:2), PC (18:0/22:5), PC (20:4/0:0), PC (20:4/18:0), PC (20:4/18:1), PC (20:4/20:2), PE (18:2/0:0), and SM (d18:0/16:0).

**Conclusions:** These results indicated a close relationship between PCs, PEs and SMs and cardiac damage mechanisms during development of DCM. The therapeutic effects of berberine on DCM are partly caused by interferences with PCs, PEs, and SMs metabolisms.

## Introduction

Diabetes mellitus (DM) is among the most prevalent and morbid chronic diseases, and the worldwide prevalence of diabetic people is predicted to dramatically increase from 415 million to 642 million by 2040 (Boles et al., 2017; Ingelfinger and Jarcho, 2017; Ruiz et al., 2018). In China, there also be a rapid increase in the prevalence of DM associated with recent economic development, and the current diabetic subjects were ~113.9 million, and a further 493.4 million persons with prediabetes (Xu et al., 2013; Chan et al., 2014). And the type 2 DM that closely related to obesity is the main contributor to the increase of DM prevalence (Ma et al., 2017).

Diabetes mellitus and prediabetes status increase all cause and cardiovascular mortality (Huang et al., 2017). Diabetic cardiomyopathy (DCM) is considered to be a distinct clinical entity independent of concomitant macro- and microvascular disorders (Huynh et al., 2014; Jia et al., 2018), and occurs in the absence of coronary artery disease, valvular disease, and other conventional cardiovascular risk factors, which could induce direct damage to myocardium and lead to heart failure (Loffroy et al., 2009; Trachanas et al., 2014; Jia et al., 2018). Various potential molecular and cellular mechanisms are involved in the pathophysiological process of DCM (Ruiz et al., 2018), such as disturbances in myocardial energy utilization, calcium signaling pathway, as well as mitochondrial dysfunction (Palomer et al., 2013).

The metabolic disturbances in DCM are mainly characterized by triglycerides (TGs) accumulation in the myocardial tissue, excessive lipid oxidation, and reduced glucose utilization (Bakshi et al., 2015), which may lead to the exceeding oxidative stress, mitochondrial dysfunction and apoptosis of cardiomyocytes (Palomer et al., 2013; Bakshi et al., 2015). In addition, the lipidomic profiling extends far beyond detection of total cholesterol (TCH) and TGs, which is becoming increasingly useful to clarify the interactions between lipid metabolism, diet, and metabolic diseases (Meikle and Summers, 2017).

Berberine is a kind of isoquinoline alkaloids that can be extracted from various plants, such as Coptis chinensis (Chinese goldthread), Berberis vulgaris (barberry), and Phellodendron amurense (Amur corktree), which is widely used in traditional Chinese medicine (Cicero and Baggioni, 2016). The current clinical and experimental research have suggested a potential effect of berberine on regulation of lipid and glucose homeostasis, as well as inhibiting inflammation and cancer growth (Wang et al., 2018). Oral administration of berberine could improve hypercholesterolemic patients and reduce serum TCH, TG, and low-density lipoprotein (LDL)—cholesterol (Kong et al., 2004). Berberine could also improve nonalcoholic fatty liver disease patients and alter circulating ceramides (Chang et al., 2016). The lowering lipid effect of berberine was related to the activation of adenosine monophosphate (AMP)-activated protein kinase (AMPK) pathway (Jiang et al., 2016) and regulation of LDL receptor (LDLR) post-transcription, as well as ameliorating abnormal lipid deposition and reducing oxidative stress (Sun et al., 2017). However, little is known about the effect of berberine on lipidomic profiling in DCM subjects (Li et al., 2015).

The recent studies in DCM animal models suggested the important role of phospholipids (e.g., phosphatidylcholine and phosphatidylethanolamine) in the development of cardiac dysfunction and remodeling of DCM (Dong et al., 2017). We also found that berberine could protect against cardiac dysfunction induced by high caloric diet via promoting glucose transport and inhibiting cardiac lipid accumulation (Dong et al., 2011). The purpose of this study was to observe the dynamic pathogenesis changes of DCM and the effect of berberine on changes of myocardial lipidomic profiles in DCM rats, in order to explore novel targets for further research.

## Materials

### Chemicals and reagents

Streptozotocin (STZ), berberine chloride, and metformin were obtained from Sigma-Aldrich (St Louis, MO, USA). The STZ was dissolved in 0.1 M citrate buffer (pH = 4.5), and berberine was suspended in 0.5% sodium carboxymethyl cellulose (CMC) solution. Liquid chromatography tandem mass spectrometry (LC-MS)-grade methanol, chloroform, and acetonitrile were obtained from Thermo Fisher Scientific (Pittsburgh, PA, USA). Biochemical kits of plasma samples were purchased from Nanjing Jiancheng Bioengineering Institute (Nanjing, China) (Dong et al., 2011).

### Animal care

Male Sprague-Dawley rats [Grade II, certificate No. SCXK (jing) 2012-0001], weighing 180 ± 20 g, were purchased from Beijing Vital River Laboratory Animal Technology Co., Ltd. (Beijing, China). All animals were maintained under specific pathogen free conditions. Regular housing temperatures were maintained between 21 and 23°C with a 12 h light−12 h dark cycle. Water and various diets (regular chow diet or HSFD) were given to animals *ad libitum*. All studies involving animals were in accordance with ethics standards of the Animal Care and Welfare Committee of Beijing University of Chinese Medicine [Certificate No. BUCM-04-2015090201-3008].

Three 100 rats were randomly divided into five groups, with 60 rats in each group. Rats in the control group (CON) were given with regular chow diet and 0.5% sodium CMC (Dong et al., 2011). Other rats were fed high-sucrose and high-fat diet (HSHFD, 2920 Kcal/kg) provided by Ke'ao Cooperation Co., Ltd. (Beijing, China), and i.p. injected with 30 mg/kg of streptozotocin (STZ) following a 12 h fast at the fifth week of HSFD feeding to induce diabetic cardiomyopathy (DCM). Seventy-two hours following STZ injection, fasting blood glucose (FBG) levels were determined and the rats with high FBG (≥11.1 mmol/L) were recruited for further research. In addition, rats in treated groups were i.g. administered with berberine at 10, 30 mg/kg and metformin at 200 mg/kg, respectively. And rats in DCM model group were just orally given with vehicle.

### Dynamic assessment of cardiac function

Parameters of cardiac function were observed at 4 time points. After administered with berberine and metformin for 4, 10, 16, and 22 weeks, respectively, the rats were intraperitoneally anesthetized with 35 mg/kg pentobarbital sodium following a 12 h fast. A 20 G catheter was placed in the left ventricle via the right common carotid artery for detection of cardiac function, i.e., left ventricular end diastolic pressure (LVEDP), and left ventricular systolic pressure (LVSP). Data were detected by MP150 system (BIOPAC Systems, Ins., CA, USA).

### Dynamic assessment of blood lipids

Following detection of cardiac function at each time point, the whole blood was collected from the right carotid artery of rats, and transferred to tubes with anticoagulant (sodium heparin). Plasma samples were prepared by centrifuging whole blood at 2,000 g for 10 min. Levels of plasma TCH and TG were determined by ultraviolet spectrophotometric approach according to the manufacturer's protocol.

### Measurement of the whole heart mass and left ventricular mass

After blood collection, the rats were euthanized by cervical dislocation (Reis et al., 2005). The hearts were excised and weighed. And the heart weight index (BWI) and left ventricular weight index (LVWI) were calculated, respectively (Bai et al., 2018).

BWI = heart weight (HW) / body weight (BW).

LVWI = left ventricular weight (LVW) / body weight (BW).

### UPLC/QTOF/MS analysis

The ultra-high-performance liquid chromatography coupled to a quadruple time of flight mass spectrometer (UPLC/Q-TOF/MS, Waters MS Technologies, Manchester, UK) was applied for data collection. And the procedures were performed as described (Dunn et al., 2011; Want et al., 2013). Freeze samples of myocardial tissue were thawed at room temperature, and 100 mg of each sample was accurately weighed and homogenized in homogenization tubes by a Speed Mill Plus (Analytik Jena, Jena, Germany). One milliliter of chloroform/methanol (3:1) were added to tissue containing in the 1.5-mL centrifuge tube (Axygen MCT-150-C, Corning, NY, USA) and thoroughly mixed on a vortex mixer for 30 min, and then the protein precipitate was pelleted in a centrifuge at 4°C and 12,000 rpm for 10 min. After centrifuge, 400 μL of subnatant was carefully collected and dried in a Savant™ SpeedVac™ High Capacity Concentrators (Thermo Fisher, Pittsburgh, USA). And then 400 μL of isopropyl alcohol/acetonitrile (1:1) was added into each tube and dissolved in ultrasound. The dissolved matter was centrifuged at 12,000 rpm for 10 min, and the supernatant (100 μL) was transferred to a vial insert (200 μL) for liquid chromatography-mass spectrometry analysis.

For C_18_ separation, mobile phase A was acetonitrile/water (60/40) and mobile phase B was isopropanol/ acetonitrile (90/10), and both A and B contained 0.1% formic acid and 10 mM ammonium acetate. The gradient conditions for reversed phase C_18_ separation were shown in Table 1.

**Table 1:** The gradient conditions for reversed phase C_18_ separation for lipids.

**Time (min)**	**A (v%)**	**B (v%)**
0	80	20
2	70	30
5	55	45
6.5	40	60
12	35	65
14	15	85
17.5	0	100
18	0	100
18.1	80	20
19.5	80	20

A Waters Acquity UPLC CSH C_18_ column (2.1 × 100 mm, 1.7 μm) was operated at 45°C and 300 μL/min flow rate. And the injection volume was 2 μL. Samples were analyzed in positive and negative electrospray ionization (ESI) using Waters xevo G2 QTOF Mass Spectrometer. And conditions of ESI^+^ and ESI^−^ were showed in Table 2.

**Table 2:** Analysis condition of positive and negative electrospray ionization.

**Parameter**	**ESI^+^**	**ESI^−^**
Capillary voltage	3,200 V	2,300 V
Sampling cone	30 V	30 V
Desolvation temperature	400°C	400°C
Desolvation gas flow	800 L/h	800 L/h
Cone gas flow	30 L/h	40 L/h
Source temperature	120°C	120°C

### Statistical methods

All of the MS data were processed by Progenesis QI software (Nonlinear Dynamics, Newcastle, UK). And the steps that carried out to conduct a sample analysis, included imputing raw data, peak alignment, picking, and normalization to produce peak intensities for retention time (*t*_R_) and mass-to-charge ratio (m/z) data pairs (Saigusa et al., 2018). The range of automatic peak picking was 0.7 ~19 min. Databases including our database containing more than 600 metabolite standards and the online databases such as LIPID MAPS (http://www.Lipidmaps.org/tools/index.html) were used to identify the lipid metabolites (Saigusa et al., 2016). For example, in positive mode the ion of m/z 544.34034 (*t*_R_, 1.3903 min) was speculated as C_28_H_50_NO_7_P by analyzing the elemental composition and the fractional isotope abundance (Qian et al., 2017). Then, this metabolite was identified as PC (20:4/0:0) after comparing the tandem mass spectrometry (MS/MS) data with Lipid Standards MS/MS spectra in LIPID MAPS.

A multivariate analysis was performed using the SIMCA 14.1 software (Meikle et al., 2014) (Umetrics AB, Umea, Sweden). An unsupervised model of principal component analysis (PCA) with unit variance scaling was used to assess the holistic metabolome alterations among groups and monitor the stability of this study. A supervised model of orthogonal projection to latent structures discriminant analysis (OPLS-DA) with unit variance scaling was performed to maximize the distance between groups and identify important variables with an important contribution to the classification according to the variable important in the projection (VIP) values. The permutation test was conducted for 200 times to evaluate the risk of over-fitting for the OPLS-DA model.

Additional data are shown as mean ± SD. In each experiment, *n* defines the number of rats. Statistical significance between multiple groups was evaluated by one-way analysis of variance (ANOVA) followed by least significant difference (LSD) *post-hoc* tests using SPSS version 17.0. *P* < 0.05 was considered as statistically significant.

## Results

### Berberine improved cardiac diastolic function in DCM rats

**Figure 1:**
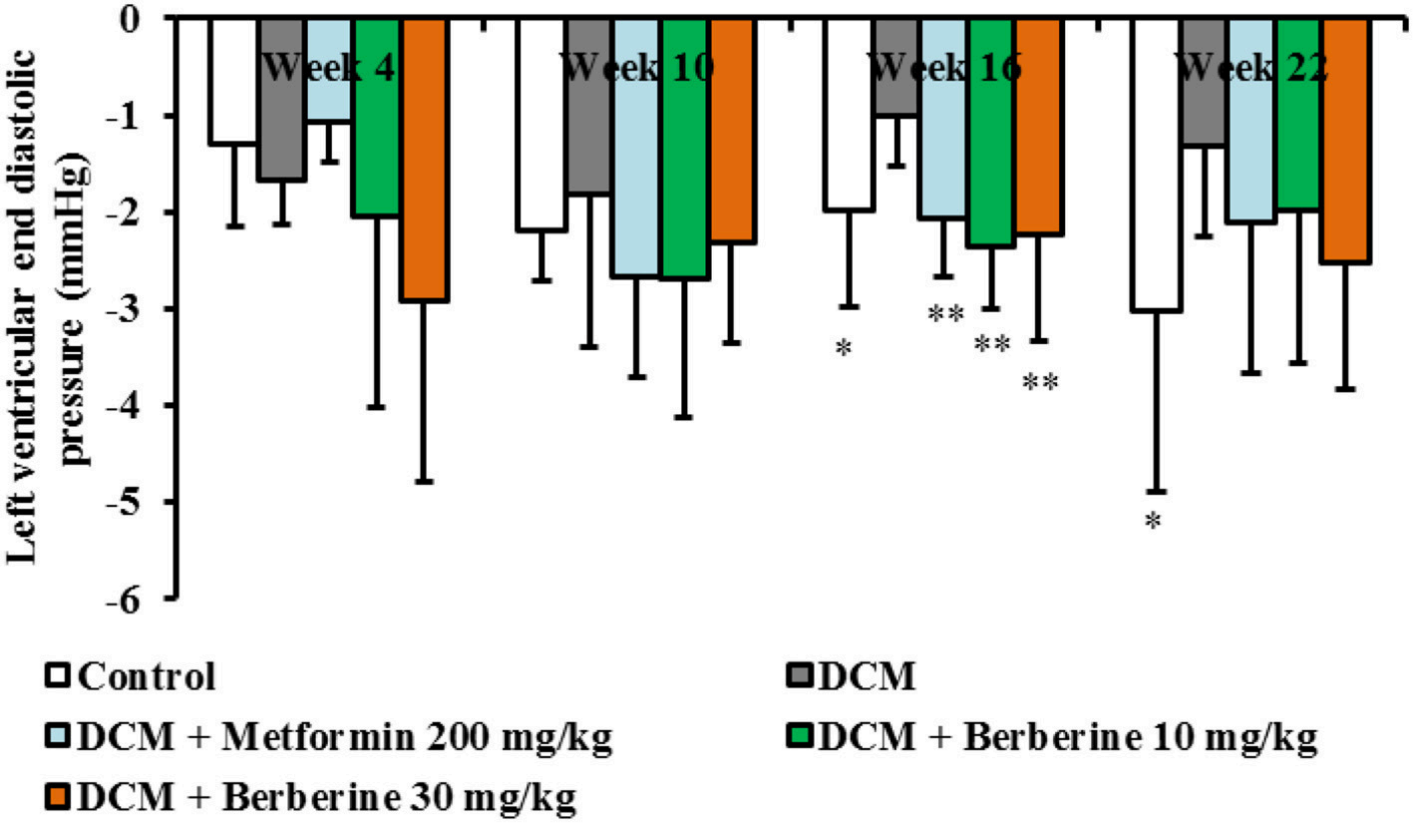
Berberine improved cardiac diastolic function in DCM rats. Diabetic cardiomyopathy (DCM) model rats were established by feeding with high-sucrose and high-fat diet (HSHFD) consisting of regular diet (66.5%), lard (10%), sucrose (20%), cholesterol (2.5%), and bile salt (1%) and intraperitoneally (i.p.) injected with 30 mg/kg of streptozotocin (STZ) at the fifth week. Seventy-two hours after STZ injection, berberine at the dosage of 10, 30 mg/kg, and metformin at 200 mg/kg were orally given to DCM rats, respectively, for consecutive 4, 10, 16, and 22 weeks. The dynamic changes of cardiac function were measured by invasive left ventricular catheterization in anesthetized rats. DCM, diabetic cardiomyopathy. Data were shown as mean ± SD, with *n* = 7~12. Statistical significance was determined by one-way analysis of variance (ANOVA) followed by least significant difference (LSD) *post-hoc* test. *P < 0.05, **P < 0.01 vs. DCM group.

In this study, the dynamic changes of cardiac function in rats were observed at week 4, 10, 16, and 22, respectively. When compared with the normal rats, LVEDP values in DCM rats increased from week 10 to week 22 and showed significant changes at week 16 (*P* < 0.05) and week 22 (*P* < 0.05), respectively. In addition, only after 16-week administration with berberine at 10, 30 mg/kg and metformin at 200 mg/kg, respectively, values of LVEDP showed remarkable reduction (*P* < 0.01), when compared with the DCM rats (Figure 1). These results indicated that berberine could protect against cardiac diastolic dysfunction, and the most effective duration is with 16-week administration.

### Berberine improved cardiac systolic function in DCM rats

**Figure 2:**
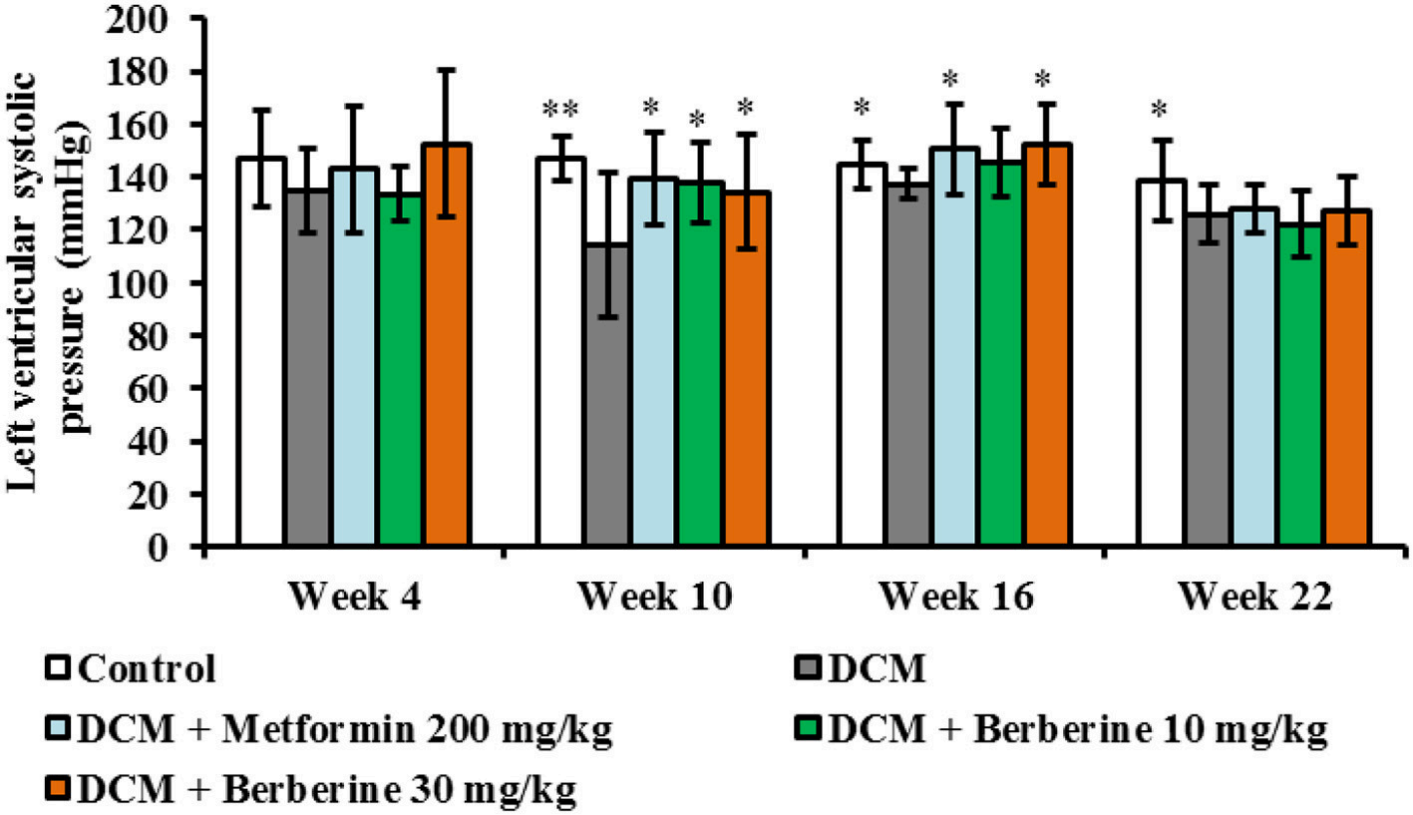
Berberine improved cardiac systolic function in DCM rats. DCM, diabetic cardiomyopathy. Data were shown as mean ± SD, with *n* = 7~12. Statistical significance was determined by one-way ANOVA followed by LSD *post-hoc* test. *P < 0.05, **P < 0.01 vs. DCM group.

When compared with control rats, values of LVSP were significantly decreased at week 10 (*P* < 0.01), week 16 (*P* < 0.05), and week 22 (*P* < 0.05). After administration with berberine at 30 mg/kg and metformin at 200 mg/kg, respectively, LVSP levels were increased significantly at week 10 and week 16 (*P* < 0.05) compared to DCM group (Figure 2). These results indicated that berberine could protect against cardiac systolic dysfunction, and the most effective duration is with 10–16 weeks of administration.

### Berberine decreased the ratios of HW/BW and LVW/BW in DCM rats

**Figure 3:**
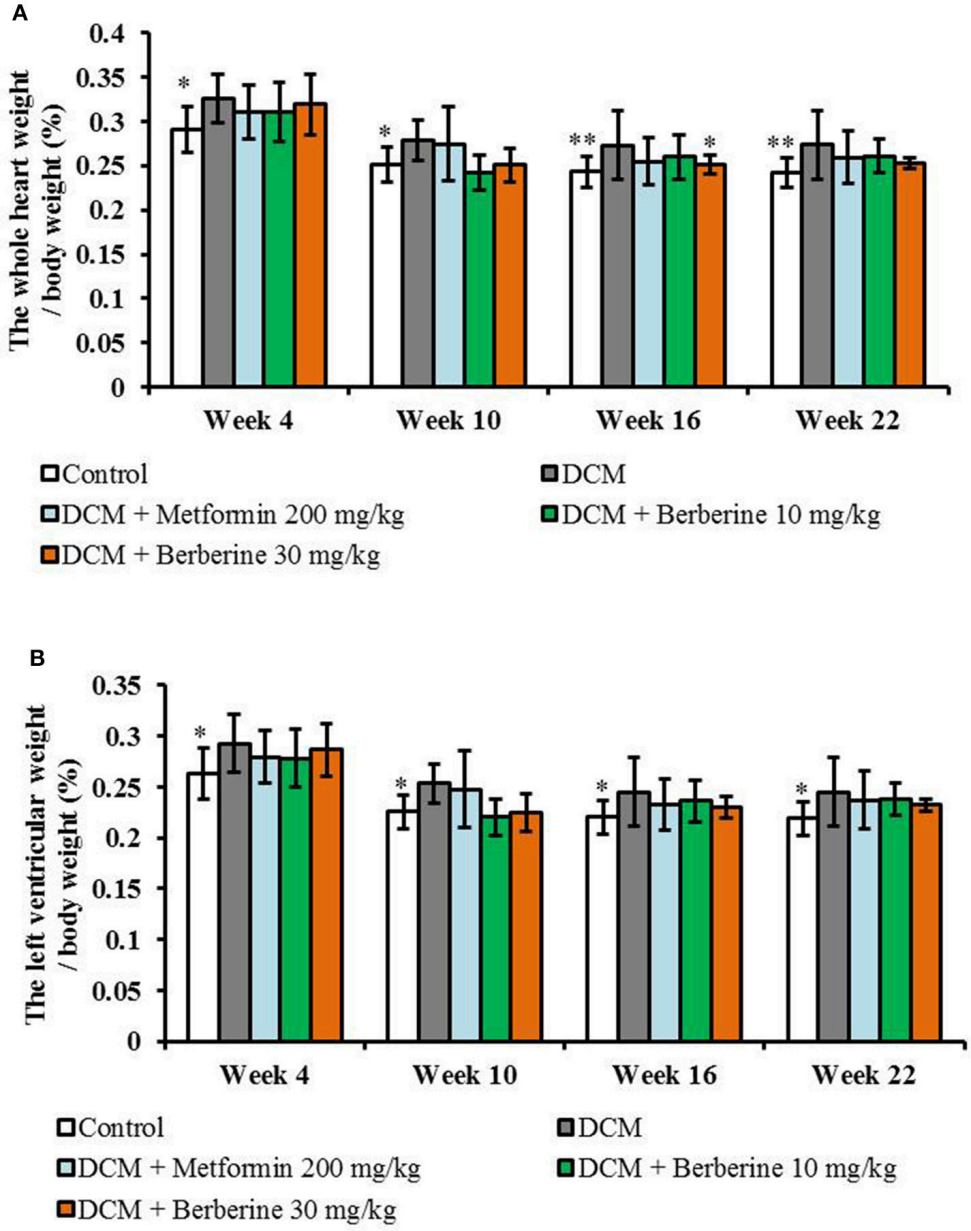
Berberine decreased the heart weight index and left ventricular weight index in DCM rats. **(A)** Heart weight index: the whole heart weight / body weight. **(B)** Left ventricular weight index: the left ventricular weight / body weight. DCM, diabetic cardiomyopathy. Data are presented as mean ± SD, with *n* = 7~12. Statistical significance was determined by one-way ANOVA followed by LSD *post-hoc* test. *P < 0.05, **P < 0.01 vs. DCM group.

When compared with the control rats, values of both LVW/BW and HW/BW were remarkably increased from week 4 to week 22 (*P* < 0.05 or *P* < 0.01) in DCM model rats, which indicated a cardiac hypertrophy status. Berberine at 30 mg/kg caused a significant reduction in HW/BW at week 16 (*P* < 0.05), when compared with DCM rats (Figure 3).

### Blood lipid parameters

**Figure 4:**
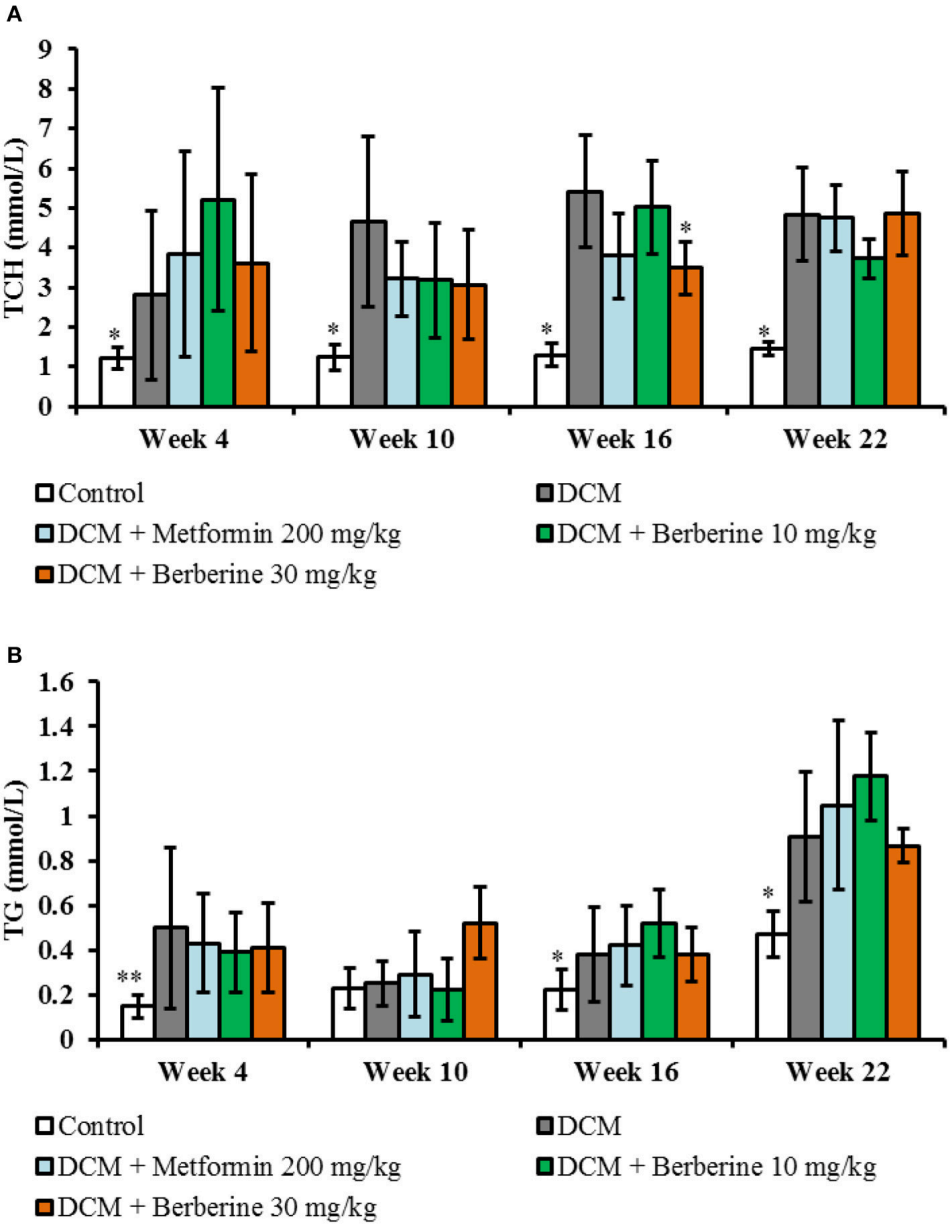
Blood lipid parameters. **(A)** Dynamic changes of total cholesterol. **(B)** Dynamic changes of triglyceride. TCH, total cholesterol; TG, triglyceride; DCM, diabetic cardiomyopathy. Data are presented as mean ± SD, with *n* = 7~12. Statistical significance was evaluated by one-way ANOVA followed by LSD *post-hoc* test. *P < 0.05, **P < 0.01 vs. DCM model group.

Levels of plasma TCH and TG of DCM rats were increased significantly from week 4 to week 22 (*P* < 0.05 or *P* < 0.01). Berberine 30 mg/kg caused a significant reduction in TCH levels at week 16 (*P* < 0.05), when compared with DCM rats (Figure 4).

Taken in concert, all above experimental results manifested protection effects of berberine on DCM rats. Berberine could protect against cardiac diastolic and systolic dysfunction, as well as cardiac hypertrophy, and the most effective duration is around 16-week administration. Therefore, the samples at this time point were selected for the subsequent lipidomic profiles analysis.

### Lipidomic profiles analysis of myocardial tissue

Metabolic profiles of the myocardial tissue were acquired by UPLC/Q-TOF/MS approach in ESI^+^ and ESI^−^ modes. Totally, 66390 positive ion peaks and 9030 negative ion peaks were obtained.

**Figure 5:**
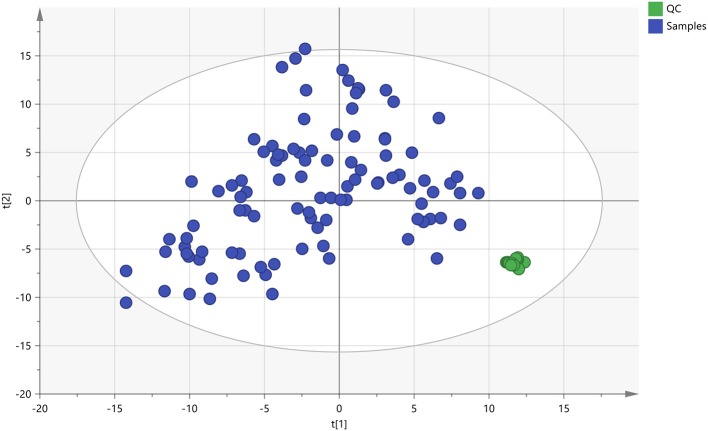
The PCA scores plot of QC and On-test Samples. Quality control (QC) samples were applied to exhibit the stability of the liquid chromatography-mass spectrometer (LC-MS) system. The cluster of the quality control samples in the principal component analysis scores scatter plot showed a satisfactory stability and repeatability of this lipidomic analysis approach. The colors display the subjects from different groups. 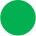: QC, quality control; 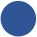, Test samples.

The quality control (QC) samples were used to monitor the stability and performance of LC-MS system and the reproducibility of the samples. The pretreatment of QC samples was in the same manner as the test samples. In this study, five QC samples of myocardial tissue were inserted at regular intervals (6~8 samples) into the run sequence in order to assess the repeatability of data (Want et al., 2013). The features were selected according to their coefficients of variation (CVs) with quality control, and features with CVs over 15% were eliminated (Saigusa et al., 2016). As shown in Figure 5, the cluster of the QC samples in the principal component analysis (PCA) scores scatter plot exhibited stability and repeatability of this lipidomic analysis system (Xu et al., 2017).

### Identification of potential biomarkers

**Figure 6:**
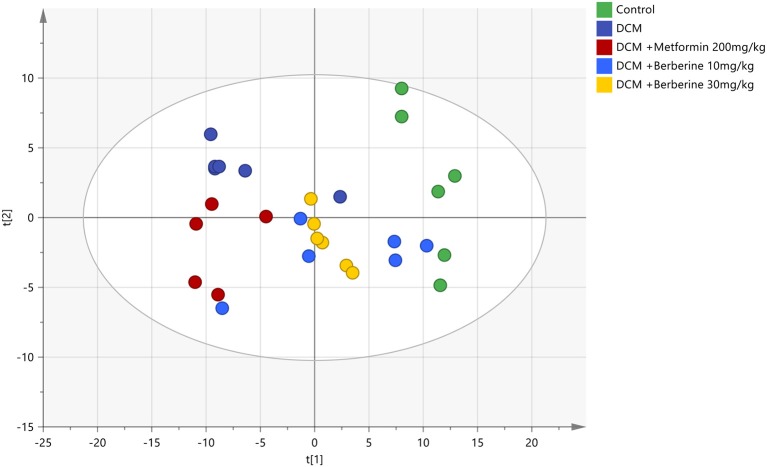
The PCA scores plot of five groups. DCM, diabetic cardiomyopathy. The colors display the subjects from different groups. 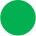, Control; 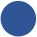, DCM; 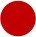, DCM + Metformin 200 mg/kg; 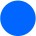, DCM + Berberine 10 mmg/kg; 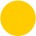, DCM + Berberine 30 mg/kg.

The PCA was applied to analyze all acquired observations in order to investigate the global lipidomics metabolism variations. The lipid metabolic phenotypes based on all imported samples could be classified by the approach of PCA method (Xu et al., 2017). An overviews of all samples in the data and a clear grouping trend (*R*^2^X, 0.861; *Q*^2^, 0.752) between the control group, DCM group, DCM +Metformin 200 mg/kg group, DCM + Berberine 10 mg/kg group and DCM + Berberine 30 mg/kg group could be observed. The DCM group *vs*. control group exhibited an improved separation. This observation indicated that DCM status may disturb the metabolism of the lipids, when compared to control group. Berberine at 10, 30 mg/kg and metformin at 200 mg/kg showed interferences in the DCM rats, although the trajectory of the drug-treated groups did not return to the normal state (Figure 6).

**Figure 7:**
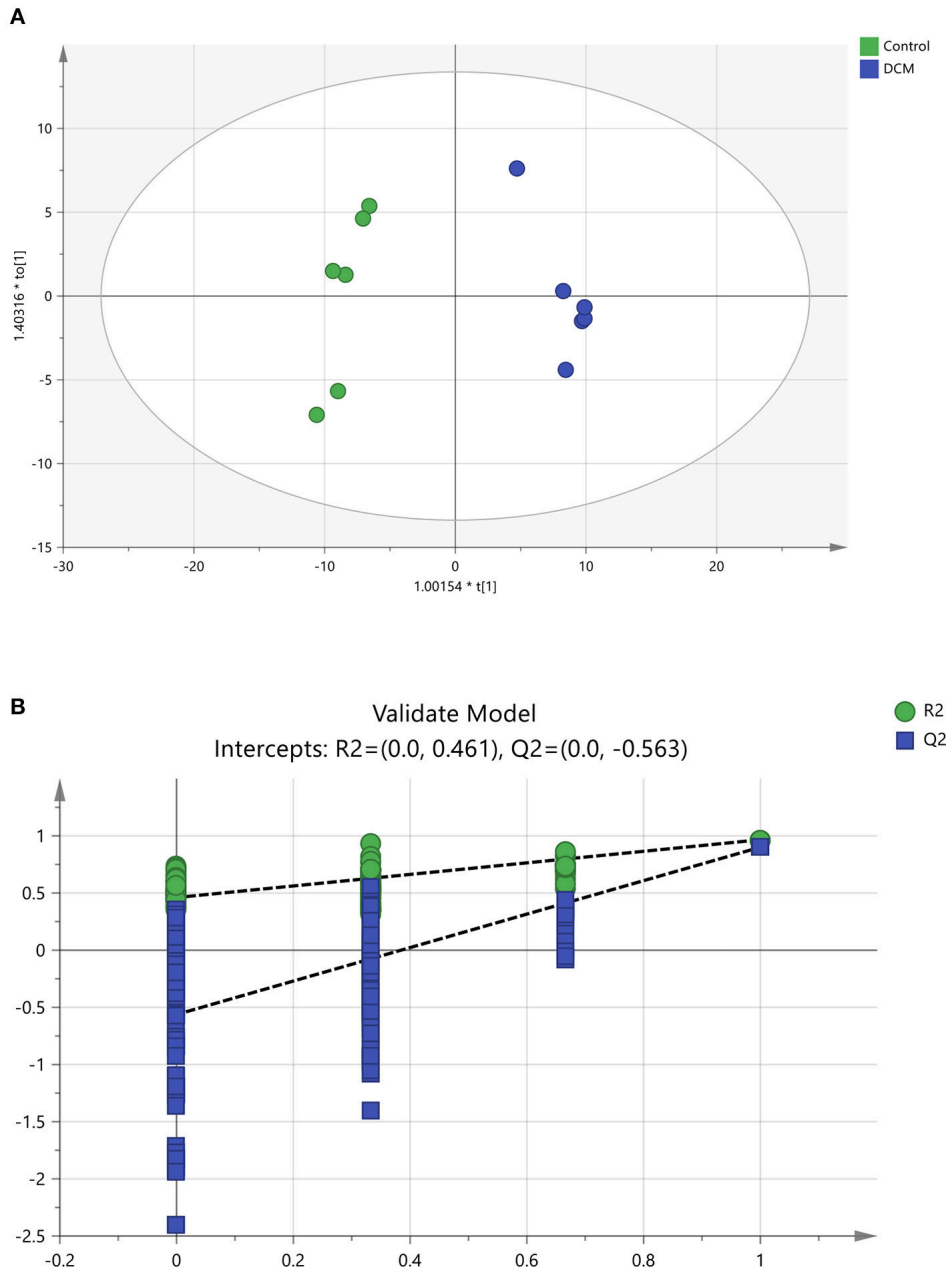
The OPLS-DA scores scatter plot and validation plot. **(A)** The orthogonal projection to latent structures discriminant analysis (OPLS-DA) scores scatter plot obtained from the diabetic cardiomyopathy (DCM) group vs. control group. **(B)** Validation plot for OPLS-DA model. The permutation test was conducted for 200 times to evaluate the risk of over-fitting for the OPLS-DA model. DCM, diabetic cardiomyopathy. **(A)** 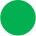, Control, 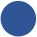, DCM model group; **(B)** 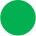, *R*^2^, 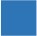, *Q*^2^.

To further confirm the certain lipids used as selective and sensitive biomarkers for pathogenesis of diabetic cardiomyopathy, the OPLS-DA approach was used to compare the changes of lipid metabolites between the DCM and the control rats (Xu et al., 2017). A clear separation of the control rats and the DCM rats was observed as demonstrated by OPLS-DA scores scatter plot (Figure 7A). In the OPLS-DA model, the cumulative *R*^2^Y and *Q*^2^ are 0.966 and 0.901, respectively. No over-fitting was observed according to results of chance permutation (Figure 7B). Afterwards, the significant changed lipid metabolites of the DCM group compared to the normal group were filtered out based on variable important in the projection (VIP) values (VIP > 1) and *t*-test (*P* < 0.05).

Subsequently, total 17 potential lipids biomarkers of DCM models, including 12 PCs, 3 PEs, and 2 SMs were further studied (Table 3).

**Table 3:** Identification of potential biomarkers related to development of diabetic cardiomyopathy rats.

**No**.	***t*_R_ (min)**	**Mass-to-charge (m/z) ratios**	**Metabolites**	**DCM vs. CON**			**MET 200** vs**. DCM**		**BER 10 vs. DCM**		**BER 30 vs. DCM**	
				**VIP values**	***P*-values**	**FC values**	***P*-values**	**FC values**	***P*-values**	**FC values**	***P*-values**	**FC values**
1	12.0796	786.60079	PC(18:0/18:2)	3.006781	1.32E-07	3.0068	0.327604	1.0807	0.013098	0.7619	0.027139	0.6058
2	1.5160	520.34032	PC(18:2/0:0)	2.690564	0.000507	2.6906	0.555070	1.1357	0.016675	0.5969	0.025931	0.5989
3	3.0185	466.32977	PE(P-18:0/0:0)	3.347361	1.62E-06	3.3474	0.307410	0.8457	0.005106	0.7163	0.450433	0.9335
4	1.5517	478.29289	PE(18:2/0:0)	2.142614	0.022380	2.1426	0.767972	1.0863	0.029045	0.4881	0.024280	0.4366
5	8.7803	705.59059	SM(d18:0/16:0)	3.805205	1.21E-06	3.8052	0.245808	0.8385	0.000135	0.5423	0.000020	0.3671
6	8.3575	729.59056	SM(d18:1/18:1)	2.043793	5.34E-05	2.0438	0.163162	0.8610	0.603399	0.9569	0.416094	0.9196
7	8.1461	706.53886	PC(16:0/14:0)	3.403970	3.14E-06	0.2938	0.000664	0.5270	0.609048	1.0346	0.796824	1.4075
8	10.8241	734.57009	PC(16:0/16:0)	2.064071	2.48E-06	0.4845	0.011311	0.7546	0.394074	0.9302	0.746697	1.0889
9	8.7446	782.56980	PC(16:0/20:4)	3.218626	3.24E-05	0.3107	0.197687	0.6865	0.048580	1.3616	0.211812	1.5514
10	11.8139	836.61668	PC(18:0/22:5)	2.477047	0.000951	0.4037	0.144162	0.5809	0.018528	1.6424	0.074504	2.3522
11	1.3903	544.34034	PC(20:4/0:0)	4.333849	7.64E-05	0.2307	0.736446	0.8593	0.017967	1.8425	0.077411	3.1387
12	11.6239	810.60078	PC(20:4/18:0)	2.534677	0.000301	0.3945	0.327411	0.7201	0.007459	1.7659	0.128390	1.7546
13	9.0317	808.58519	PC(20:4/18:1)	2.578941	0.000242	0.3878	0.218952	0.6849	0.035153	1.4237	0.197382	1.6132
14	7.3106	806.56949	PC(20:4/18:2)	2.010264	0.001226	0.4974	0.153197	0.6732	0.153344	1.2372	0.917770	1.0087
15	10.8527	834.60065	PC(20:4/20:2)	3.423473	0.000324	0.2921	0.124597	0.5275	0.010437	1.8687	0.100724	2.6785
16	6.8407	830.56954	PC(20:4/20:4)	2.833827	8.79E-05	0.3529	0.031543	0.5630	0.052507	1.3728	0.225158	1.6431
17	9.2859	740.52268	PE(20:4/16:0)	1.919220	8.79E-05	0.5210	0.059134	0.7180	0.207394	1.1066	0.781551	1.1054

*t_R_, retention time; VIP, variable important in the projection; FC, folder change; PC, phosphatidylcholine; PE, phosphatidylethanolamine; SM, sphingolipid; CON, control group; DCM, diabetic cardiomyopathy model group; MET 200, DCM + Metformin 200 mg/kg group; BER 10, DCM + Berberine 10 mg/kg group; BER 30, DCM + Berberine 30 mg/kg group*.

**Figure 8:**
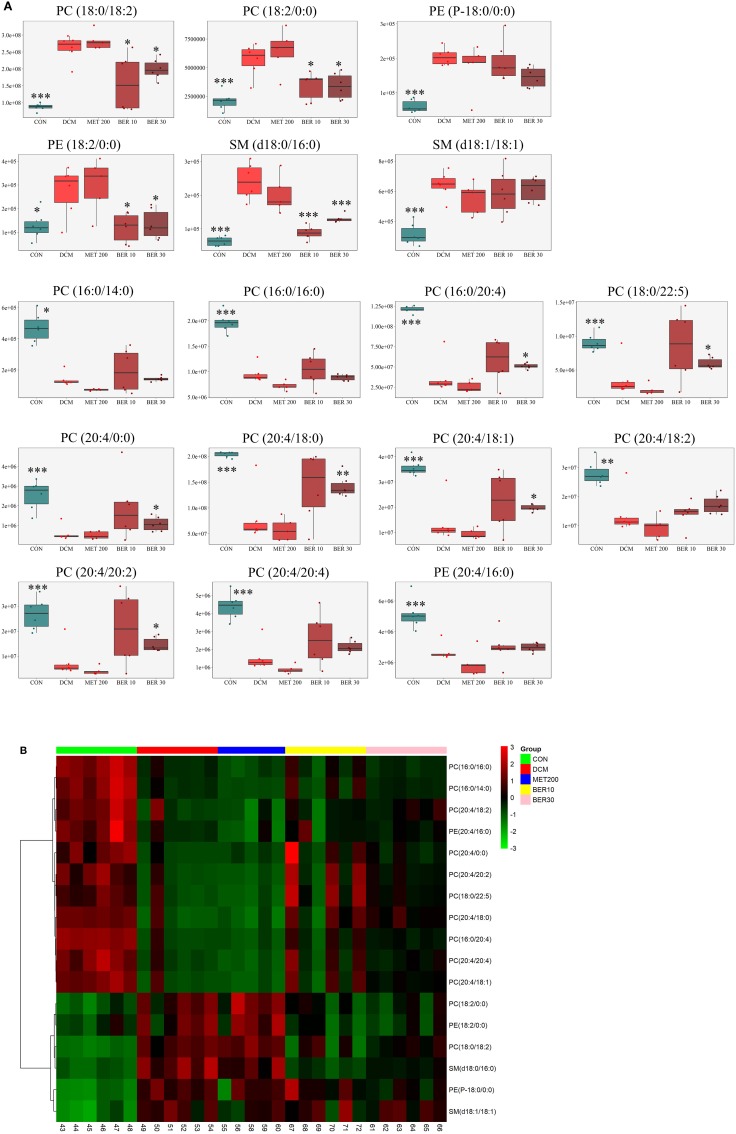
Identification of potential biomarkers related to diabetic cardiomyopathy development. **(A)** Metabolic changes of lipids in the five test groups. **(B)** Heat map. Only metabolites with VIP > 1 and *P* < 0.05 were selected, and different shades of color present the concentration (red, black, and green presented the high, normal, and low concentration, respectively). CON, control group; DCM, diabetic cardiomyopathy model group; MET 200, DCM + Metformin 200 mg/kg group; BER 10, DCM + Berberine 10 mg/kg group; BER 30, DCM + Berberine 30 mg/kg group; PC, phosphatidylcholine; PE, phosphatidylethanolamine; SM, sphingolipid. Statistical significance was evaluated by one-way ANOVA followed by LSD *post-hoc* test. *P < 0.05, **P < 0.01, and ***P < 0.001 vs. DCM group.

The changed levels and names of selected biomarkers were shown in Figure 8. Specifically, the lipid levels of 2 PCs, 2 PEs, and 2 SMs, including PC (18:0/18:2), PC (18:2/0:0), PE (P-18:0/0:0), PE (18:2/0:0), SM (d18:0/16:0), and SM (d18:1/18:1) were significantly up-regulated in the DCM rats, when compared with normal rats (*P* < 0.05 or *P* < 0.001). Meanwhile, lipid levels of 10 PCs and 1 PE, including PC (16:0/14:0), PC (16:0/16:0), PC (16:0/20:4), PC (18:0/22:5), PC (20:4/0:0), PC (20:4/18:0), PC (20:4/18:1), PC (20:4/18:2), PC (20:4/20:2), PC (20:4/20:4), and PE (20:4/16:0) were remarkably reduced in the DCM rats, when compared with normal rats (*P* < 0.05 or *P* < 0.001).

After administered with berberine, lipid levels of 8 PCs, 1 PE, and 1 SM were significantly altered and exhibited a normal-level tendency (*P* < 0.05), i.e., PC (18:0/18:2), PC (18:2/0:0), PC (16:0/20:4), PC (18:0/22:5), PC (20:4/0:0), PC (20:4/18:0), PC (20:4/18:1), PC (20:4/20:2), PE (18:2/0:0), and SM (d18:0/16:0).

## Discussion

Dyslipidemia is a major contributor to the pathological process of DCM, and the main therapeutic methods involve lowering lipid (Meikle and Summers, 2017). In the present study, we explored the association between altered lipidomic profiling and cardiac dysfunction in the diabetic rats, as well as the regulation of berberine.

Current lipid parameters commonly used clinically are including TG, TCH, high-density lipoprotein and low-density lipoprotein cholesterol, which provide a relative narrow snapshot of the dynamic processes of lipid metabolism (Wong et al., 2013; Meikle et al., 2014). The application of lipidomics analyzed approach has been greatly expanding our understanding of the extent and complexity of lipid dysregulation in diabetes and its complications (Meikle and Summers, 2017). Lipidomics has been used to explore the physiological functions of lipid molecules (Hu and Zhang, 2018), and it was also applied to discover the biomarkers for diagnosis and therapy of metabolic disease (Meikle et al., 2014; Hu and Zhang, 2018).

The use of HPLC increases the sensitivity for low abundance lipids and their metabolites (Zhao et al., 2015). Identification of lipid metabolites in this method is achieved on the basis of searching for accurate mass-to-charge ratios from databases of known lipids and further tandem mass spectrometry based experiments (Nygren et al., 2011; Meikle et al., 2014). This approach has been applied for the analysis of plasma and serum in depicting the pathophysiological mechanism of disease (Laaksonen et al., 2006; Yetukuri et al., 2011; Meikle et al., 2014). Furthermore, when investigating the molecular mechanisms of diseases, and the metabolic changes induced by pathophysiological stress are considered to be more concentrated in some specific organs of localized diseases such as DCM, the use of related organs in this platform are useful (Nam et al., 2017; Hu and Zhang, 2018).

Our previous research indicated that berberine at 30 mg/kg orally administered for 6 weeks could attenuate cardiac dysfunction and cardiac hypertrophy in rats induced by feeding high-sucrose/fat diet plus i.p. injection with streptozotocin (30 mg/kg) through alleviating cardiac lipid accumulation and promoting glucose transport (Dong et al., 2011). Then, we used UPLC/Q-TOF/MS analyzed approach to explore the changes of myocardial lipidomic profiles in diabetic rats. And the results manifested that the cardiac lipid metabolites have remarkable correlation with changes of cardiac function and heart mass ratio (Dong et al., 2017).

In this study, we observed dynamic changes of cardiac functions, heart mass ratios and blood lipids at week 4, 10, 16, and 22, respectively. And the results demonstrated that berberine could protect against cardiac diastolic and systolic dysfunctions, as well as cardiac hypertrophy, and the most effective duration is with 16-week administration. In addition, 17 potential biomarkers of phosphatidylcholines (PCs), phosphatidylethanolamine (PEs) and sphingolipid (SMs) of DCM induced by HSFD/STZ have been identified. Furthermore, our results manifested that the perturbations could be partly reversed by berberine intervention, i.e., PC (18:0/18:2), PC (18:2/0:0), PC (16:0/20:4), PC (18:0/22:5), PC (20:4/0:0), PC (20:4/18:0), PC (20:4/18:1), PC (20:4/20:2), PE (18:2/0:0), and SM (d18:0/16:0).

Many phospholipid and sphingolipid metabolites have been proved to be important components linking obesity to type 2 DM and cardiovascular diseases (Meikle and Summers, 2017). The major components of the phospholipids involve the phosphatidylcholines (PCs) and phosphatidylethanolamines (PEs).

As the major constituents of the biologic membranes, the phospholipids can prevent the stability of cell membrane degeneration and promote recovery via improving cell membrane, protecting cells and mitochondria membrane against injury, promoting synthesis of lipoproteins, and increasing membrane fluidity and enzyme activities (Morrison et al., 2012; Zhang et al., 2014). Phospholipids also allow selected molecules to diffuse or move across the cell membrane into cells (Bishop and Bell, 1988; Zhang et al., 2014). And the membrane phospholipids are also the reservoir of lipid mediators and signaling molecules such as arachidonic acid (AA), prostaglandins, inositol triphosphate, endocannabinoids, and diacylglycerol (Lamari et al., 2013).

Besides important role involved in many cell processes, the phospholipids are the probable modulators of cardiac muscle insulin resistance rather than diacyglycerol or triacyglycerol (Quehenberger et al., 2010). Lots of evidences indicated the significance of impaired phospholipid-mediated signaling systems in DCM, heart failure and cardiac hypertrophy (Tabbi-Anneni et al., 2003). Also, *in vitro* and *in vivo* studies have confirmed the association between dysregulated phospholipid metabolism and cardiac lipotoxicity in DCM (Lim et al., 2011).

A plasma lipidomic study in humans indicated a relationship of PC species with risk measures of metabolic diseases, and PC (32:1) and PC (38:3) were associated with a high risk of metabolic syndrome (Kulkarni et al., 2013). There were 45 glycerophospholipid and sphingolipid (SM) species observed reduced in the coronary artery disease (Sutter et al., 2016). A population-based research indicated that serum PC (18:2/0:0) was associated with several cardiovascular disease-risk factors in adolescents (Syme et al., 2016). Serum lipid metabolites PC (18:0/18:2) and PC (16:0/14:0) were observed reduced from pre-diabetes to diabetes (Zeng et al., 2017). A clinical observation showed a persistent lipid signature with higher levels of plasma TGs, and diacylphospholipids as well as lower levels of alkylacyl phosphatidylcholines in progress to type 2 DM (Suvitaival et al., 2018). And plasma SM (d18:1/18:1) was significantly decreased in type 1 diabetic patients relative to controls (Sorensen et al., 2010). The myocardial phosphatidylinositol mass increased by 46%, and 1-stearoyl-2-arachidonoyl phosphatidylethanolamine decreased by 22% in diabetic rats induced by STZ (Han et al., 2000). The *easily shocked* (*eas*) mutant heart in *Drosophila* exhibited defects in cardiac physiology, including reduction in diastolic and systolic diameters, as well as heartbeat length and the absolute volume output, and exhibited increased concentrations of TG, and low levels of PE, which suggested that lipid metabolism and cardiac function could be regulated by phospholipid homeostasis (Lim et al., 2011).

And it has been confirmed that the maintenance of the balance of the PC: PE ratio has important health potentials (Korematsu et al., 2017). Blood-based lipidomic studies in humans showed a strong association of reduced PC: PE ratio with obesity, type 2 DM and prediabetes (Meikle et al., 2013; Weir et al., 2013; Henstridge et al., 2015).

In terms of diabetic cardiomyopathy, the cardiomyopathic status such as cardiac hypertrophy, fibrosis, and cell death induced by both hyperglycemia and cardiac metabolic imbalance can lead to abnormal Ca^2+^ transients and contractile activity. The cellular ion homeostasis imbalance and changes of contractile proteins can impair the excitation-contraction coupling, and potential of autonomic responsiveness and autonomic neuropathy. Also, the structural abnormalities occur (Tappia, 2007). The lipid metabolites, especially the main components of cardiomyocytes membrane such as phosphatidylcholine and phosphatidylethanolamine are closely related to intracellular ion homeostasis, which may contribute to the structural and functional alterations in the diabetic heart (Ussher et al., 2016; Meikle and Summers, 2017). Insulin treatment of diabetic rats could restore the species profile of phosphatidylethanolamines and overcorrect the changes in molecular species of phosphatidylcholines (Vecchini et al., 2000), which suggested that the abnormal in molecular species of glycerophospholipids may play an important role in membrane dysfunction and defective contractility of the diabetic heart (Vecchini et al., 2000; Tappia, 2007).

## Conclusions

Our results indicated a close relationship between PCs, PEs, and SMs and cardiac damage mechanisms during development of DCM. And the therapeutic effects of berberine on DCM are partly due to interferences with PC, PE, and SM metabolisms.

## Availability of data and supporting materials

The datasets used and analyzed during the current study available from the corresponding author or the first author on reasonable request.

## Author contributions

JS and SD designed experiments. RZ, SZ, LC, ZC, and SD performed experiments. FS and RZ performed UPLCQ-TOF/MS analysis. LT and SD conducted statistical analysis. SD wrote this paper. And all authors read and approved the final manuscript.

### Conflict of interest statement

The authors declare that the research was conducted in the absence of any commercial or financial relationships that could be construed as a potential conflict of interest.
